# Effects of Experienced Disgust on Morally-Relevant Judgments

**DOI:** 10.1371/journal.pone.0160357

**Published:** 2016-08-02

**Authors:** Bunmi O. Olatunji, Bieke David Puncochar, Rebecca Cox

**Affiliations:** Vanderbilt University, Nashville, TN, United States of America; Universita degli Studi di Perugia, ITALY

## Abstract

Although disgust has been implicated in moral judgments, the extent to which the influence of disgust on moral judgment is distinct from other negative affective states remains unclear. To address this gap in knowledge, participants in Study 1 were randomized to a disgust (hand submersion in imitation vomit), discomfort (hand submersion in ice water), or neutral (hand submersion in room temperature water) affect condition while moral judgments of offenses were simultaneously assessed. The results showed that participants in the discomfort condition made the most severe moral judgments, particularly for moderate offenses. To examine if disgust may have more of an effect on some moral violations than others, participants in Study 2 were randomized to similar affect inductions while judgments of purity and non-purity offenses were simultaneously assessed. The results showed that those who had their hand submerged in imitation vomit recommended harsher punishment for purity violations relative to moral violations unrelated to purity. The opposite was true for those who submerged their hands in ice water, whereas punishment ratings for purity and non-purity violations did not significantly differ for those who submerged their hands in room temperature water. The implications of these findings for further delineating the specific role of experienced disgust in moral decision-making are discussed.

## Introduction

Recent research suggests that decisions about the moral severity of various acts are primarily guided by affective processes [1,2]. Disgust in particular is thought to have a strong association with morality [3,4] and has received much attention recently in the research literature [1,5,6]. Although disgust is traditionally viewed as an emotion that functions to protect the organism from contact with potentially contaminating substances, thereby promoting disease avoidance [7], it is also thought to regulate moral behavior [8,5] by signaling that objects, behaviors, or persons are to be avoided to maintain social order. Thus when disgust is experienced when evaluating a moral violation, the emotion may be used as information that the violation is more severe. Inducing disgust may make some stored knowledge more accessible (e.g., reasons to be disgusted by rape are spotlighted) and this more accessible information then has a greater impact on subsequent preferences [9]. Consistent with this view, Wheatley and Haidt [1] found that hypnotically induced disgust results in more severe moral judgments. A recent series of studies also found that for those sensitive to internal sensations (i.e., high private body consciousness), induced disgust led to increased moral severity in judgments of morally relevant behaviors, but not in non-moral ones [10].

The majority of the experimental evidence implicating disgust in moral decision-making has compared judgments after disgust induction with judgments after a neutral induction [1]. However, such paradigms leave open the possibility that the influence of disgust on moral judgment is mainly one of negative affect, rather than disgust specifically. In response to such concerns, some studies have compared disgust with sadness, and some support for the specificity of disgust in moral judgments has been observed from such studies. For example, Schnall and colleagues [10] found that participants thought disgusting (e.g., eating one’s dog) and non-disgusting (e.g., falsifying one’s resume) moral transgressions were more severe after disgust priming via a movie clip than after sadness priming. Horberg and colleagues [6] also found that participants who had been primed with disgust via a movie clip, compared to those primed with a sadness clip, reported more perceived moral wrongness and rightness of purity violations (e.g., being sexually promiscuous) and virtues (e.g., maintaining a healthy body), respectively. In a more recent study by Moretti and di Pellegrino [11], participants rejected unfair offers significantly more often after disgust priming than after sadness or neutral priming. Rejecting an unfair offer can be seen as a behavior response based on values of fairness and reciprocity [12]. From these findings, one might conclude that disgust is distinct from other forms of negative affect in its ability to influence morally relevant decisions.

The ethics of divinity describe a person as a spiritual entity that connects the self to a higher force. Divinity also prescribes the body as sacred, making it important to maintain purity [13]. This suggests that disgust may be linked to violations of purity and not necessarily other types of moral violations. Although the disgust-purity link has received some measure of empirical support [6], it has not been a consistent finding. For example, Royzman and colleagues [14] found that anger, but not disgust, may be the principal emotional response to moral transgressions irrespective of the normative content involved. More specifically, examination of projected responses to pathogen-free violations of the divinity code revealed little evidence of disgust-related phenomenology (nausea, gagging, loss of appetite) or action tendency (desire to move away), but strong evidence of anger-linked desire to retaliate. This anger-dominant attribution pattern remained intact when the impious act being judged was the judge's own. This finding highlights the need for further research to clarify the nature of the moral violations that are uniquely linked to disgust.

Another important point for consideration is that current studies examining the unique role of inducing disgust on moral judgements have largely compared disgust and sadness. Such studies have also done so using paradigms in the laboratory where disgust (or sadness) is induced during a first phase of the experiment, and its influence on moral judgments is tested in a second, later, phase. While these phases typically follow each other closely, there nevertheless is opportunity for the induced emotions to dissipate. This is important because some emotions may dissipate more slowly than others, and in turn have a stronger influence on subsequent morality judgments. Indeed, Olatunji, Forsyth and Cherian [15] found disgust to be a particularly “sticky” emotion that is fairly resistant to extinction. Scherer and Walbott [16] do report that disgust experiences have relatively short duration, but this is presumably after the stimulus is removed. Two-phase paradigms used in prior research may thus lead to erroneous conclusions about the robust role of disgust in moral judgment because this is the emotion that is still being experienced when rating moral transgressions, while sadness may have already dissipated. Mood induction procedures for sadness typically involve watching a sad film clip or recalling a sad life event, often while listening to sad music, and the mood they produce is typically experienced for less than 10 min [17,18]. Unlike disgust, the short-lived nature of experimentally induced sad mood in normal participants makes it difficult to assess the effects of sadness in more than one task at a time [19]. This observation suggests that an experimental paradigm where affect induction and moral judgment occur simultaneously may prove to be very informative regarding the role of disgust in moral judgment.

If sadness and disgust do dissipate at a similar rate, different moral judgments after disgust versus sadness priming may still not warrant the conclusion that disgust is robustly associated with moral judgments. Instead, it may be more parsimonious to conclude that sadness and disgust have a differential influence on moral judgments, and that disgust—compared to sadness—more strongly informs moral decisions. That is, sadness may be less likely to influence judgment of unrelated moral offenses in part because the emotion is often associated with deliberate, systematic processing and a reduced reliance on heuristics [20]. Such detail-oriented processing may prevent sadness that is unrelated to a moral offense from influencing judgments about the offense. Accordingly, sadness may not be the ideal control comparison by which strong inferences can be made regarding the extent to which disgust influences moral decisions. Research directly comparing disgust with other types of negative affect that are not associated with systematic processing may allow for stronger inferences.

The current study aimed to address limitations of prior research by examining the extent to which the experience of disgust, compared to the experience of discomfort, influences moral judgments that are made during the affective induction rather than after the induction. Discomfort, in this study, refers to physical uneasiness that was evoked with a cold pressor task, and was used as a comparison to disgust because of its clear negative valence, but lack of distinct secondary appraisals. Consistent with prior research indicating a strong role for disgust in moral decision-making, it was predicted that ratings of immorality regarding moral transgressions would be higher among those induced to experience disgust relative to those in the neutral and discomfort induction condition. However, if the increased severity of moral judgments associated with disgust is an artifact of the negative valence associated with the emotion, then ratings of immorality in the discomfort and disgust condition are expected to be significantly higher than those in the neutral condition, but not differ significantly from one another.

## Study 1 Method

This research was approved by the Vanderbilt University IRB Committee. Specifically, all participants gave their written informed consent and all procedures of the study (including the written informed consent, and the autonomy of each participant to stop at any point during the study) were kept in line with the IRB regulations.

### Participants

Seventy-seven undergraduate students (82% female, 69% Caucasian) participated in exchange for course credit.

### Materials

The *Disgust Scale-Revised* (DS-R; [21]; modified by Olatunji et al. [22]) is a 25-item scale that measures disgust sensitivity across the domains of core, animal reminder, and contamination disgust. The *DS-R* had an alpha coefficient of .84 in the present study. The DSR was administered to be sure there were no group differences in baseline disgust sensitivity.

#### Moral Transgressions

Twelve one-sentence descriptions of three levels of moral transgression (non-offenses, moderate offenses, severe offenses) were presented to participants in random order (see Appendix A). Non-offenses (*N* = 4) described behavior that is not morally wrong (e.g., F.W. went for a walk in the park). Moderate offenses (*N* = 4) described behavior that is morally questionable, but not extremely immoral (e.g., K. D. lied to a good friend). Severe offenses (*N* = 4) described extreme wrongdoings (e.g., G.S. murdered two people in their own home). These 12 offenses were selected from a larger pool of offenses on the basis of pilot data.

#### Morality Ratings

Participants were asked to rate how morally wrong they found each transgression on a scale from 0 (not morally wrong at all) to 7 (extremely morally wrong). They were also asked to indicate how negative they found each of the transgressions on a scale from 0 (not at all negative) to 7 (extremely negative).

### Affect Induction

Affect in this study was induced via submersion of the participants’ left hand (in a thin plastic glove) into one of three liquids (depending on the condition to which they were randomly assigned) while rating each of 12 moral transgressions. In the disgust condition, this liquid consisted of the following ingredients: cream of mushroom soup, cream of chicken soup, black beans, and chopped-up pieces of fried gluten. This recipe was partially based on a recipe for imitation vomit developed by Tsao and McKay [23]. The liquid in the negative condition consisted of ice water (50 degrees Fahrenheit, or 10 degrees Celsius), which was kept constant by adding additional ice cubes throughout the experiment. Finally, in the neutral condition, the liquid was lukewarm water (80 degrees Fahrenheit, or 27 degrees Celsius). All liquids were kept in white, four-gallon containers.

### Procedure

After completing the DS-R, participants were randomized to one of three affect inductions. In the disgust condition, participants placed their gloved hand in a container filled with imitation vomit. They were instructed that the substance was actually vomit but not asked to report how protected they felt the gloves were. Participants were informed that we were interested in their ability to multitask. Participants were asked to hold their hand in the vomit while making their ratings of immorality and negativity for each moral transgression. After rating a given transgression, participants removed the glove from their hand, discarded it, and put on a new glove to prevent habituation. In the negative condition, participants submerged their gloved hand in a container filled with ice water while making their ratings. Immediately following completion of the ratings for a given transgression, participants placed their hand in another container with lukewarm water (27 degrees Celsius or 80 degrees Fahrenheit) for 2 minutes to raise their hand temperature before submerging it in ice water again to rate the next transgression. In the neutral condition, participants placed their gloved hand in lukewarm water while rating the moral transgressions. The container was visible to the participants in all three conditions.

For all participants, the time between finishing ratings for one transgression and starting ratings for the next transgression was two minutes. This time allowed for raising hand temperature (in the negative condition) and helped prevent carry-over effects from one transgression to the next. After all 12 transgressions were rated, participants indicated how negative and disgusted their task made them feel, as well as how much discomfort they experienced during this task on a scale from 0 (not at all) to 7 (extremely). This study was approved by an Institutional Review Board.

### Results

#### Manipulation Check for Pre-Existing Group Differences

A univariate analysis of variance (ANOVA) was conducted with condition (disgust, discomfort, neutral) as the independent variable and scores on the DS-R as the dependent variables. A univariate ANOVA was conducted with condition (disgust, discomfort, neutral) as the independent variable and average scores on the DS-R as the dependent variable. The analysis failed to yield a main effect of condition [*F* (2, 74) = 1.20, *p >* .05, partial η^2^ = .03] suggesting that scores on the DS-R for those in the disgust condition did not significantly differ from those in the discomfort or the neutral condition (see Table 1 for means and standard deviations). The gender distribution for the disgust (% Female = 76.9), discomfort (% Female = 92.0), the neutral (%Female = 76.9) also did not significantly differ, χ² = 2.58, *p* = .275.

**Table 1:** Means (M) and Standard Deviations (SD) for Scores on the Disgust Scale-Revised (DS-R).

		Condition	
	Neutral	Disgust	Discomfort
	*M (SD)*	*M (SD)*	*M (SD)*
**Study 1**			
DS-R	2.21 (.44)	2.11 (.61)	2.35 (.57)
**Study 2**			
DS-R	2.27 (.44)	1.96 (.56)	2.36 (.62)

#### Affect Induction Manipulation Check

**Fig 1:**
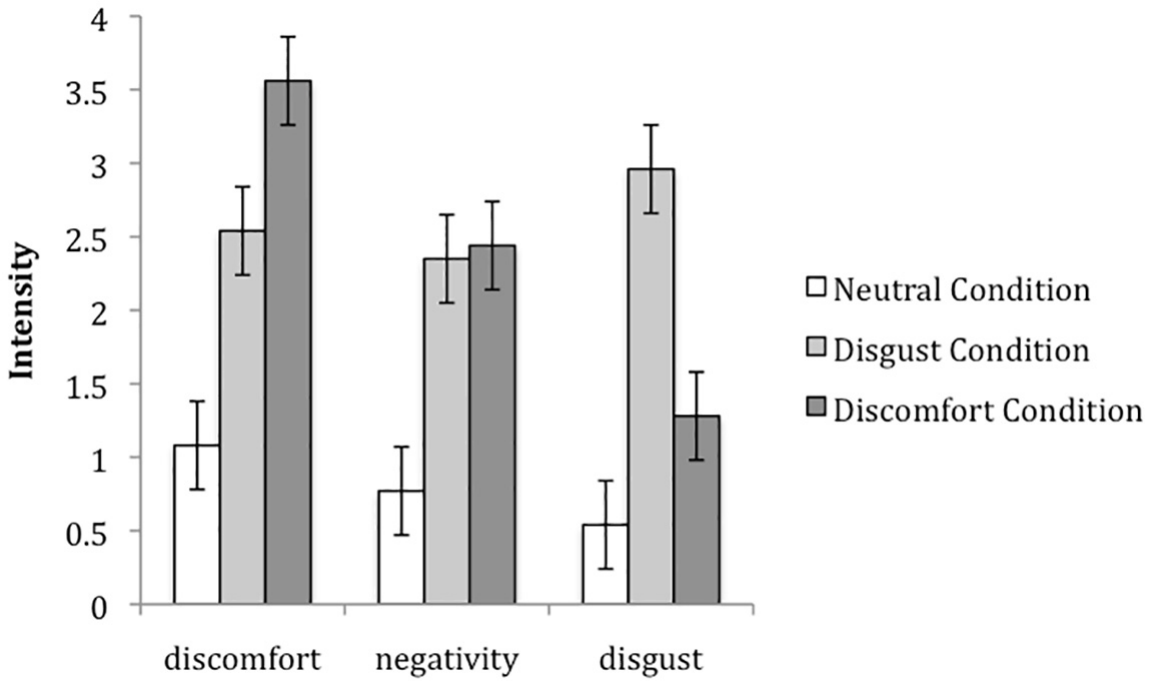
Disgust, Negativity and Discomfort ratings in the Disgust, Discomfort and Neutral Affect Condition in Study 1.

A 3 (Condition: disgust, discomfort, neutral) X 3 (Emotion rating: disgust, negativity, discomfort) mixed-factor ANOVA yielded significant main effects of condition, *F*(2, 74) = 14.34, *p* < .001, partial η^2^ = .28 and emotion rating, *F*(2, 148) = 13.31, *p* < .001, partial η^2^ = .15. These main effects were qualified by a significant condition X emotion rating interaction [*F*(2, 148) = 12.90, *p* < .001, partial η^2^ = .26]. To examine this interaction, disgust, discomfort and negativity ratings were entered in a multivariate ANOVA with condition as the independent variable. The analysis revealed a main effect of condition for disgust [*F*(2, 74) = 17.58, *p* < .001, partial η^2^ = .32], discomfort [*F*(2, 74) = 16.69, *p* < .001, partial η^2^ = .31] and negativity [*F*(2, 74) = 8.47, *p* < .001, partial η^2^ = .19]. Pairwise comparisons revealed that participants reported significantly more disgust in the disgust condition (*M* = 2.96 *SD =* 1.87) than in the discomfort condition (*M* = 1.28, *SD =* 1.60, *p* < .001) or in the neutral condition (*M* = 0.54, *SD =* .91, *p* < .001), and the latter two did not significantly differ. Furthermore, participants reported significantly more discomfort in the discomfort condition (*M* = 3.56, *SD =* 1.78) than in the disgust condition (*M* = 2.54, *SD =* 1.77, *p* < .001) and in the neutral condition (*M* = 1.08, *SD =* .94, *p* < .001). They also reported more discomfort in the disgust than in the neutral condition (*p* = .021). Although negativity did not differ in the disgust and discomfort conditions (see Fig 1), participants reported significantly more negativity in the disgust (*M* = 2.35 *SD =* 1.70) and discomfort (*M* = 2.44, *SD =* 1.92) conditions than in the neutral condition (*M* = 0.77, *SD =* 1.24, *p*_disgust-neutral_ < .001, *p*_negative-neutral_ < .001).

#### Effect of Affect Induction on Moral Judgments

**Fig 2:**
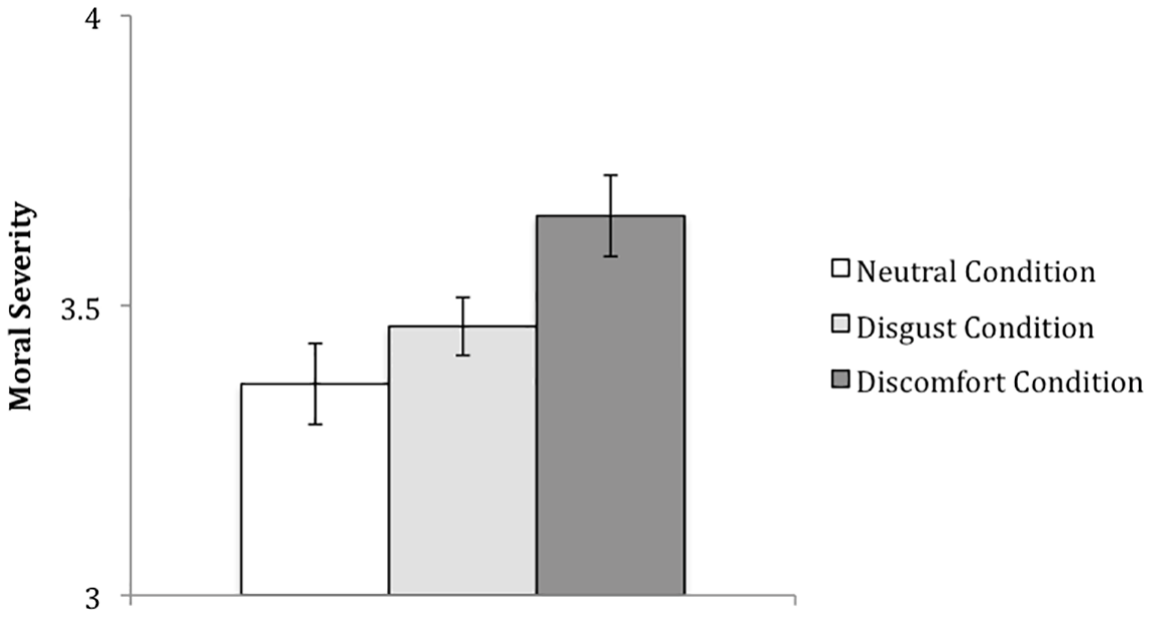
Morality Ratings for Moderate Violations per Affect Condition.

Due to the high correlation between perceived morality and negativity of the offenses (*r* = .74, *p* < .001), these ratings were combined into one score (averaging immorality and negativity). These combined scores will be referred to as “morality ratings” in what follows, and were entered in a 3 (Condition: disgust condition, discomfort condition, neutral condition) X 3 (Offense Level: non-offense, moderate offense, severe offense) mixed-factor ANOVA. The analysis revealed a main effect of condition [*F*(2,74) = 4.73, *p* = .012, partial η^2^ = .11] and offense level [*F*(2,148) = 3431.41, *p* < .001, partial η^2^ = .98]. These main effects of offense level and condition were also qualified by a significant condition X offense level interaction, *F*(4,148) = 2.84, *p* = .026, partial η^2^ = .07 (see Table 2 for means and standard deviations). To examine this interaction, morality ratings for each offense level were then entered in a multivariate ANOVA with condition (neutral, disgust, discomfort) as the independent variable. Although a significant main effect was not observed for non-offenses [*F*(2,74) = 1.66, *p* = .19, partial η^2^ = .04] and severe offences [*F*(2,74) = 1.88, *p* = .15, partial η^2^ = .05], a main effect of condition for moderate offenses was found, *F*(2,74) = 3.86, *p* = .025, partial η^2^ = .09. As shown in Fig 2, pairwise comparisons for moderate offenses showed that participants rated transgressions as more morally wrong in the discomfort condition (*M* = 4.08, *SD* = .87) than in the neutral condition (*M* = 3.42, *SD* = .99, *p* = .008), and there was a non-significant trend towards more moral severity in the discomfort condition compared to the disgust condition (*M* = 3.62, *SD* = .73, *p* = .06). No significant differences were found in immorality ratings of moderate offenses between those in the disgust and neutral condition (*p* = .40).

**Table 2:** Means (M) and Standard Deviations (SD) for Morality Ratings per Condition and Moral Offense Level.

		Condition	
	Neutral	Disgust	Discomfort
Offense Level	*M (SD)*	*M (SD)*	*M (SD)*
Non-Offense	.01 (.05)	.00 (.00)	.10 (.38)
Moderate Offense	3.42 (.99)	3.62 (.73)	4.08 (.87)
Severe Offense	6.67 (.27)	6.77 (.21)	6.79 (.24)
All Offenses	3.36 (.38)	3.46 (.28)	3.65 (.36)

#### Association between Individual Differences in Disgust and Moral Judgments

Exploratory analyses were then conducted to examine the extent to which scores on the DS-R correlated with morality ratings in the full sample. Scores on the DS-R was not significantly correlated with any of the moral judgments (*r*s range from -.04 to .10, *p*s > .37).

### Discussion

The findings of Study 1 showed that more severe judgments of immorality were made in the discomfort condition especially for moderate offenses. One explanation for this finding is that discomfort may have influenced moral judgments more so than disgust due to the greater intensity with which it was induced. That is, disgust may have not influenced moral judgments in this study because it was induced less intensely than discomfort. This *intensity disparity* observed in Study 1 may have important methodological and theoretical implications for interpreting previous research that has found that disgust influences moral judgments more so than other negative emotions. Methodologically, these findings highlight the importance of matching emotion induction conductions as closely as possible in the intensity with which the emotions are experienced. Differences in emotion intensity may reflect differences in emotional arousal. Importantly, arousal, rather than the valance of the emotion per se, may account for more variance in subsequent moral judgments [24]. Although it is clear that people are often influenced by the emotions they feel when making moral judgments, one importantly theoretical implication of this work is that it is the emotion that they feel more intensely that ultimately influences their decision. However, more research that attempts to more closely equate the intensity of emotion induction conductions is needed to text this hypothesis.

Prior research has shown that disgust is specifically associated with violations of purity and sanctity [25], and disgust priming has been found to have an influence on moral judgments about purity violations, but not on fairness violations [6]. Purity may be defined as freedom from anything that debases, contaminates, or pollutes. However, a specific effect of disgust on moral judgments about purity violations has not been a consistent finding in the literature [26,10]. When considering the available literature, it is unclear whether the null findings for disgust in this study is due to a failure to account for the observation that disgust may only influence judgments specifically about purity violations. Accordingly, a second study was conducted where participants were asked to rate moral violations of purity and those unrelated to purity during a disgust or discomfort induction that we attempted to more closely match for intensity.

To identify a disgust induction paradigm that elicited disgust with the same intensity as the ice water induction paradigm elicited discomfort, a pilot study was conducted where 15 participants (78% female, 67% Caucasian) placed their left hand, without a glove, in 50-degree ice water, 80-degree lukewarm water, room temperature imitation vomit, room temperature “real” pig intestines, and room temperature “fake” pig intestines, and rated the disgust and discomfort of each of these substances on a scale from 0 (not at all) to 7 (extremely). Both types of intestines consisted of sausage casing stuffed with a mixture of mashed potatoes, gravy, and red dye. They differed from one another only by the color and density of the stuffing, and one was labelled “real,” while the other was identified as “fake”. The textures were presented in random order and were rated twice by each participant to account for order and comparison effects, and to measure habituation that may occur due to repeated exposure. There was a significant rating (disgust, discomfort) X liquid (lukewarm water, ice water, fake intestines, real intestines, imitation vomit) interaction, *F*(4, 56) = 24.54, *p* < .001. Follow-up repeated measures ANOVAs demonstrated a main effect of texture for disgust ratings, *F*(4, 56) = 112.81, *p* < .001, as well as discomfort ratings, *F*(4, 56) = 49.57, *p* < .001. The primary aim of this pilot study was to identify emotion-induction paradigms that differed in terms of induced disgust, but not in terms of induced discomfort, and that induced disgust with the same intensity as ice water did discomfort. Pairwise comparisons showed that imitation vomit induced significantly more disgust (*M =* 5.07, *SD* = 1.29) than did ice water (*M =* .40, *SD* = .54, *p* < .001), while imitation vomit (*M =* 4.47, *SD* = 1.14) and ice water (*M =* 4.57, *SD* = 1.43, *n*.*s*.) elicited equal levels of discomfort, indicating the suited nature of these paradigms. In addition, exposure to lukewarm water elicited no disgust (*M =* 0.00, *SD* = 0.00) or discomfort (*M =* 0.00, *SD* = 0.00), motivating the use of lukewarm water in the neutral condition), or during a neutral affect induction that served as a control condition. With this design, any differences in moral judgments between the disgust and discomfort condition can more clearly be attributed to characteristics of disgust and discomfort respectively, as opposed to an intensity disparity. If intensity and negativity are primary factors in determining the influence of emotions on judgments about moral violations, no differences were anticipated in the pattern of morally-relevant judgments in the disgust and discomfort conditions. Furthermore, if disgust is particularly informative for violations of purity, an interaction was expected between condition and violation type, with disgust induction leading to more severe judgments of purity relative to non-purity violations.

Study 2 also extends the findings of Study 1 by examining the effects of experienced disgust on judgments of punishment. Although research suggests that decisions about the severity of immoral acts are primarily influenced by emotion [1,2], it remains unclear if punishment decisions are also influenced by emotions [27]. There may be reasons to predict that experienced disgust may have more of an influence on decisions about moral severity relative to decisions about punishment. Decisions about the moral wrongness of various transgressions can be experienced as a flash of affect [28], whereas decisions about punishment for those same transgressions may require more deliberate, conscious reasoning. Punishment decisions are said to be reached by first determining responsibility of the offender and then deciding on the appropriate level of punishment [29,30]. However, determination of responsibility requires some deliberation, including the assessment of culpability and intentionality. Furthermore, certainty about culpability is thought to be crucial in determining appropriate punishment [31]. The more deliberate conscious processing of information that informs decisions about punishment may make such decisions more immune to the relatively automatic influence of emotion.

## Study 2 Method

This research was approved by the Vanderbilt University IRB Committee. Specifically, all participants gave their written informed consent and all procedures of the study (including the written informed consent, and the autonomy of each participant to stop at any point during the study) were kept in line with the IRB regulations.

### Participants

Sixty-one undergraduate students (75% female, 72% Caucasian) participated in this study in exchange for course credit.

### Materials

The DS-R described in Study 1 was also administered in this study. The DS-R had an alpha coefficient of .84 in the present study. The DSR was administered to be sure there were no group differences in baseline disgust sensitivity.

#### Moral Transgressions

Due to the more pronounced effect of affective inductions for judgments about moderate moral violations as demonstrated in Study 1, only moderate violations (*N* = 20) were used in Study 2 (see Appendix B). Ten of these violations contained an element of impurity (e.g., John *urinated* on someone’s car door handle), while the other ten did not represent impurity (e.g., John lied about how many hours he worked). These transgressions were selected on the basis of a pilot study. To identify non-purity and purity violations that are moderate in severity, 20 undergraduate participants (67% female, 78% Caucasian) were asked to rate 56 moral transgressions (28 representing impurity, 28 not representing impurity), in terms of immorality, disgust and punishment on an 8-point scale ranging from 0 (not at all morally wrong / disgusting / deserving of punishment) to 7 (extremely morally wrong / disgusting / deserving of punishment). Transgressions in the purity and non-purity category were matched in terms of proportion of violations occurring toward a person (70%) or property (30%), and were all described as intentional (as opposed to accidental). Word count of non-purity transgressions was matched to that of purity transgressions. Purity (*M* = 4.60, *SD* = 1.44) and non-purity violations (*M* = 4.63, *SD* = .89) were rated as equally morally wrong, *F*(1,18) = .02, *n*.*s*., and purity violations (*M* = 5.89, *SD* = .86) were considered significantly more disgusting than non-purity violations (*M* = 3.73, *SD* = 1.17), *F*(1, 18) = 56.26, *p* < .001. Finally, purity violations (*M* = 4.11, *SD* = 1.38) and non-purity violations (*M* = 4.01, *SD* = .99) were considered equally deserving of punishment, *F*(1, 18) = .28, *n*.*s*.

#### Affect Induction

Affect was induced via submersion of participants’ left hand, without the use of a glove, in one of three liquids: imitation vomit at room temperature (disgust condition), 50-degree ice water (discomfort condition), or 80-degree lukewarm water (neutral condition). These emotion induction procedures were selected on the basis of a pilot study.

#### Morality Ratings

Participants were asked to indicate on a scale from 0 (not at all) to 7 (extremely) how morally wrong they found 0 purity violations and 10 non-purity violations. Participants were also asked to indicate how deserving of punishment they found each transgression on a scale from 0 (not at all deserving of punishment) to 7 (extremely deserving of punishment). Morality ratings of non-purity transgressions were significantly correlated with morality ratings of purity transgressions (*r* = .69, *p* < .01), punishment ratings of non-purity transgressions (*r* = .51, *p* < .01), and punishment ratings of purity transgressions (*r* = .41, *p* < .01). Morality ratings of purity transgressions were significantly correlated with punishment ratings of non-purity transgressions (*r* = .40, *p* < .01), and punishment ratings of purity transgressions (*r* = .63, *p* < .01). Punishment ratings of non-purity transgressions were also significantly correlated with punishment ratings of purity transgressions (*r* = .75, *p* < .01).

### Procedure

Participants were randomly assigned to one of the three conditions (disgust, discomfort, neutral). Once consent was obtained, participants completed the DS-R. Participants were informed that we were interested in their ability to multitask. Next, participants placed their hand in a container with a specific substance (imitation vomit, ice water, or lukewarm water). Participants were asked to indicate how morally wrong and deserving of punishment they found each of the 20 transgressions. Participants observed a prompt on the computer screen indicating that it was time to place their hand in the container. Once the hand was well submerged, the experimenter pressed a key to proceed to a screen displaying a moral violation. After three seconds, participants were asked to indicate the level of moral wrongness and deservingness of punishment in response to the moral violation. These questions were presented in random order, and there was no time limit to respond. Once each set of questions was completed, a message on the computer screen instructed participants to remove their hand from the container. At this point, the experimenter offered a paper towel to dry their hand (neutral condition); asked participants to place their hand in a container with warmer water (discomfort condition); or asked participants to wash off their hand in a container with lukewarm water, and then dry it with a paper towel (disgust condition). This procedure was employed to offer an activity between different ratings (neutral condition), to prevent habituation (disgust and discomfort condition), to prevent pain (discomfort condition), and to equate the time between rating moral transgressions (set at 1 minute). Consistent with Study 1, moral judgments were made simultaneously with the affective induction to rule out dissipation disparity as a cause for differential findings between disgust and discomfort. After completing all the ratings, participants were asked to indicate to what extent, on a scale from 0 (not at all) to 7 (extremely so), the hand submersion task made them experience disgust and discomfort. Finally, participants were debriefed and dismissed. This study was approved by an Institutional Review Board.

### Results

#### Manipulation Check for Pre-existing Group Differences

A univariate ANOVA was conducted with condition (disgust, discomfort, neutral) as the independent variable and average scores on the DS-R as the dependent variable. The analysis failed to yield a main effect of condition [*F* (2, 58) = 1.20, *p >* .05, partial η^2^ = .09] suggesting that scores on the DS-R for those in the disgust condition did not significantly differ from those in the discomfort or the neutral condition (see Table 1 for means and standard deviations). The gender distribution for the disgust (% Female = 71.4), discomfort (% Female = 80), the neutral (%Female = 75) also did not significantly differ, χ² = .40, *p* = .815.

#### Affect Induction Manipulation Check

**Fig 3:**
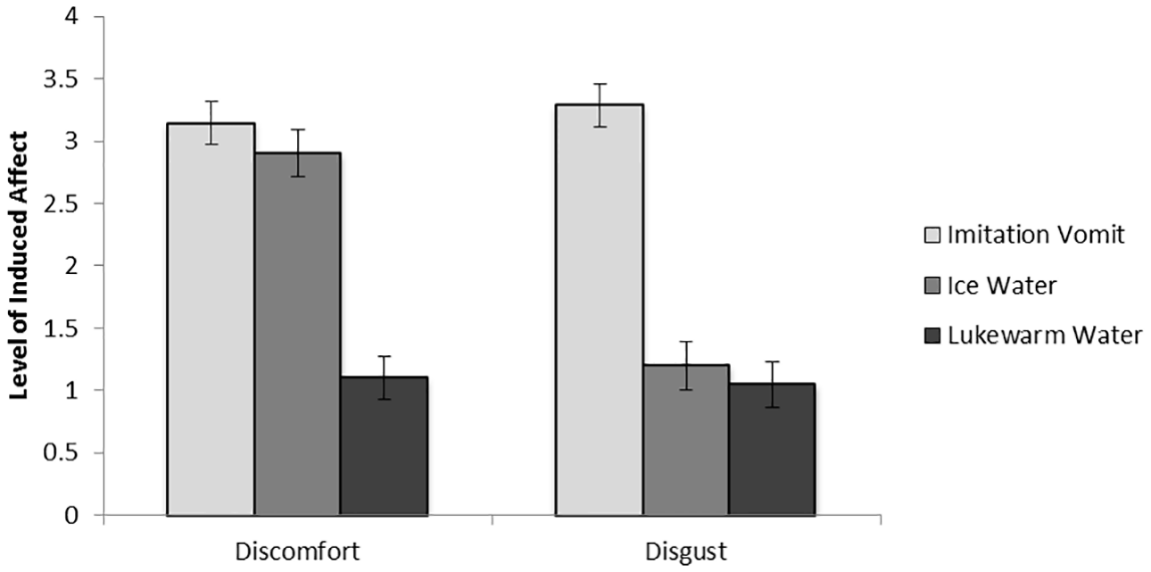
Disgust and Discomfort ratings in the Disgust, Discomfort and Neutral Affect Condition in Study 2.

To confirm that exposure to imitation vomit without a glove induced disgust to the same degree as ice water did discomfort, final disgust and discomfort ratings were entered in a 2 (rating: disgust, discomfort) by 3 (condition: disgust, discomfort, neutral) mixed-model ANOVA. This revealed a significant rating by condition interaction, *F*(2, 58) = 10.22, *p* < .001, partial η^2^ = .26. To examine this interaction, disgust and discomfort ratings were entered in a multivariate ANOVA with condition as the independent variable, revealing a main effect of condition for disgust [*F*(2, 58) = 10.85, *p* < .001, partial η^2^ = .27] and discomfort [*F*(2,58) = 10.27, *p* < .001, partial η^2^ = .26]. As shown in Fig 3, follow-up pairwise comparisons showed that participants felt significantly more disgusted when exposed to imitation vomit (*M* = 3.29, *SD* = 2.15) than when exposed to ice water (*M* = 1.20, *SD* = 1.58; *p* < .001) or lukewarm water (*M* = 1.05, *SD* = 1.32: *p* < .001), with no significant differences between the latter two. Follow-up pairwise comparisons also showed that exposure to ice water (*M* = 2.90, *SD* = 1.74; *p* < .001) and imitation vomit (*M* = 3.14, *SD* = 1.62; *p* < .001) induced significantly more discomfort than exposure to lukewarm water (*M* = 1.10, *SD* = 1.30).

#### Effect of Affect Induction on Morality

A 3 (condition: disgust, discomfort, neutral) by 2 (violation type: purity, non-purity) mixed-model ANOVA for moral wrongness ratings revealed no main effect of condition [*F*(2, 58) = .26, *p* = .76, partial η^2^ = .009] or the predicted interaction of condition by violation type [*F*(2, 58) = 2.25, *p* = .11, partial η^2^ = .07]. This suggests that morality ratings for purity and non-purity violations did not significantly differ as a function of exposure to imitation vomit, ice water, or lukewarm water (see Table 3 for means and standard deviations).

**Table 3:** Means (M) and Standard Deviations (SD) for Morality and Punishment Ratings for Purity and Non-purity Violations.

		Condition	
	Neutral	Disgust	Discomfort
	*M (SD)*	*M (SD)*	*M (SD)*
**Morality**			
Non-Purity	5.04 (.60)	4.87 (.77)	5.06 (.84)
Impurity	4.84 (.71)	4.71 (1.02)	4.51 (.89)
**Punishment**			
Non-Purity	4.41 (.77)	4.14 (.79)	4.18 (.85)
Impurity	4.45 (.79)	4.50 (.98)	3.96 (.99)

#### Effect of Affect Induction on Punishment

**Fig 4:**
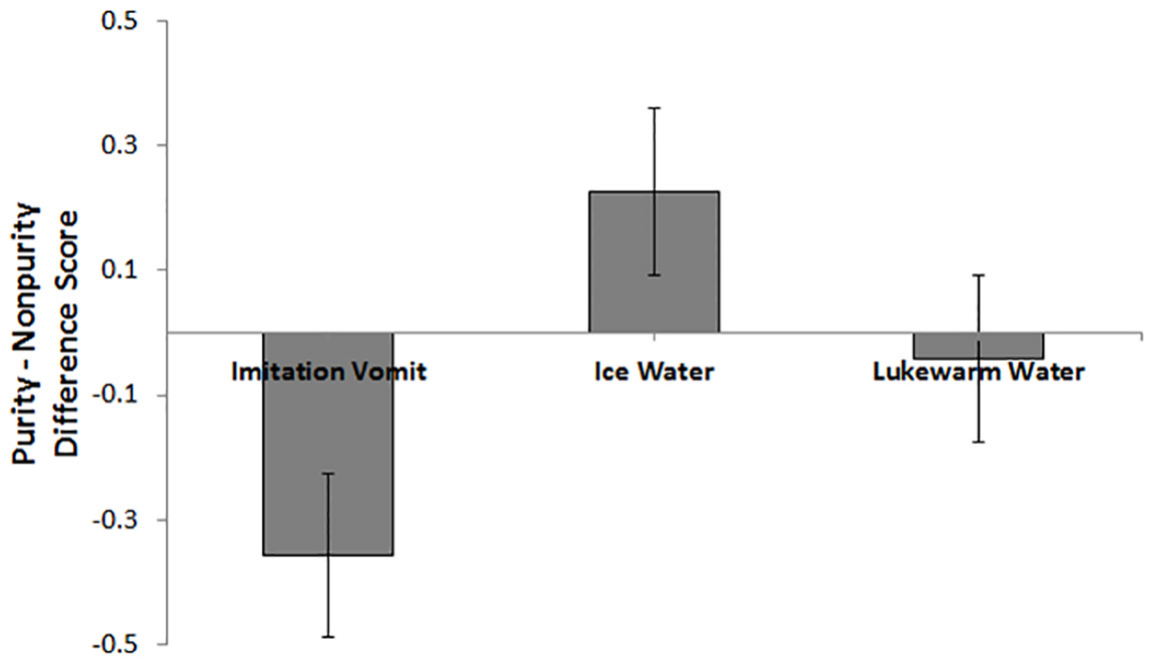
Differences in Recommended Punishment for Purity relative to Non—Purity Violations.

A 3 (condition: disgust, discomfort, neutral) by 2 (violation type: purity, non-purity) mixed-model ANOVA revealed a significant violation type by condition interaction for punishment, *F*(2, 58) = 4.88, *p* = .01, partial η^2^ = .14 (see Table 3 for means and standard deviations). To examine this interaction, difference scores were computed between punishment ratings of the non-purity and purity violations. The difference scores were then examined with a univariate ANOVA that revealed a significant main effect of condition, *F*(2, 58) = 4.88, *p* = .01. Subsequent pairwise comparisons revealed that the difference score did not significantly differ (*p* = .09) between those in the disgust condition (*M* = -.35, *SD* = .71) and neutral condition (*M* = -.04, *SD* = .57). The difference score for those in the discomfort condition (*M* = .22, *SD* = .46) and those in the neutral condition also did not significantly differ (*p* = .16). However, the difference score between those in the disgust condition and those in the discomfort condition did significantly differ (*p <* .004). Fig 4 depicts the difference scores showing that those in the disgust condition recommended more punishment for purity, relative to non-purity, violations whereas the opposite was true for those in the discomfort condition.

#### Association between Individual Differences in Disgust and Moral Judgments

Exploratory analyses were then conducted to examine the extent to which scores on the DS-R correlated with morality and punishment ratings in the full sample. The DS-R was significantly correlated with morality ratings for purity offenses (*r* = .31, *p* < .02) but not morality ratings for non-purity offenses (*r* = .23, *p* = .07). However, the correlation with purity violations was not significantly higher than that for non-purity violations. The DS-R was not significantly correlated with punishment ratings for purity (*r* = .23, *p* = .07) or non-purity offenses (*r* = .12, *p* = .34).

### Discussion

The difference (.39) between experienced disgust in the imitation vomit condition and experienced discomfort in the ice water condition in Study 2 was less than the difference (.63) between experienced disgust in the imitation vomit condition and discomfort in the ice water condition in Study 1. This suggests that the intensity of the two mood induction conditions was more comparable in Study 2 compared to Study 1. In fact, the mean level of experienced disgust in the imitation vomit condition was lower than the mean level of experienced discomfort in the ice water condition in Study 1, whereas the opposite was true for Study 2. The findings of Study 2, in which disgust and discomfort were induced with more comparable intensity, revealed no significant differences between moral severity judgments made during disgust and discomfort induction. This finding supports the notion that discomfort may have exerted a stronger influence than disgust on moral judgments in Study 1 due to the greater intensity with which it was elicited. The comparison of purity and non-purity violations did reveal a specific, albeit more limited role of disgust in punishment judgments about moral violations. Participants who had their hand submerged in imitation vomit recommended harsher punishment for moral violations of purity than for violations unrelated to purity. However, the opposite was true for those that submerged their hand in ice water.

## General Discussion

Previous research suggests that disgust informs moral judgments [1,10] and would predict more perceived immorality of moral transgressions when disgust is induced relative to other affective states. However, Study 1 found that the most severe judgments of immorality were made in the discomfort condition. This finding is inconsistent with previous research implicating disgust in moral judgment [11]. One possible explanation for the discrepant findings is differences in experimental method. Prior studies where significant effects were found for disgust, relative to sadness and neutral affect, have typically employed a two-phase approach where emotions are induced first (phase one), and their effect on moral judgments is tested later (phase two). This approach may be problematic given that laboratory induced sadness may dissipate faster than experimentally induced disgust [17,18]. In the current study, the influence of disgust on moral judgment was tested with a “one-phase” paradigm where moral judgments were assessed during the affect induction. Although future research is needed to examine the effects of different affective induction methods and the timing of such methods in relation to moral judgments, this may be an unlikely explanation for the null findings in Study 1 if disgust intensity is of any significance. Indeed, paradigms where disgust is induced first (phase one) and moral judgments are assessed later (phase two) may lend themselves to less intense disgust emotions that then inform moral judgments compared to paradigms where moral judgments are assessed simultaneously with the disgust induction.

The finding of Study 1 suggests that the intensity with which affect is induced may play an important role in moral judgments. Discomfort was induced more strongly with ice water than disgust was induced with imitation vomit in Study 1. This intensity disparity may account for moral judgments being rated as more severe for those in the discomfort condition. Interestingly, such differences in the extent to which the intended affect is induced are not unique to the current study. For example, close inspection of prior studies comparing the influence of disgust and sadness on moral judgments [10,6,11] consistently report stronger induction of disgust in the disgust condition than sadness in the sadness condition. This important difference in intensity of affective inductions may account for why disgust is reported to have a stronger influence on moral judgments than sadness in prior research, and why discomfort has a stronger influence on moral judgments than disgust in the current study.

The effects of experienced discomfort on moral judgments were also found to be driven primarily by morality ratings of moderate offenses in Study 1. Non-offenses described behavior that is not morally wrong, thus there is no reason to anticipate that affective information would be utilized to make a decision about moral severity of such offenses (i.e., floor effect). Given that severe offenses described extreme wrongdoings (i.e., ceiling effect), use of extraneous affective information may not be required to judge such violations. The ambiguity of moderate offenses may render decisions about their moral severity more difficult. Accordingly, participants may be more susceptible to influences of extraneous emotions in judging such offenses (Affect-as-information [32]).

Although the finding of Study 1 is not consistent with the claim that disgust substantially influences a range of moral judgments, evaluation of the literature does suggest that disgust may slightly influence the severity of a relevant (but narrow) class of moral judgments [33]. Consistent with this view, Horberg and colleagues [6] found that integral feelings of disgust, but not integral anger, predicted stronger moral condemnation of behaviors violating purity. With integral emotion effects, the emotion elicited by a particular event influences judgments made about that same event. Furthermore, experimentally induced disgust, compared with induced sadness, increased condemnation of behaviors violating purity and increased approval of behaviors upholding purity. Lastly, trait disgust, but not trait anger or trait fear, predicted stronger condemnation of purity violations and greater approval of behaviors upholding purity. This research suggests that disgust may be linked with moralization of the purity domain and the failure to account for this distinction in the type of moral violation may explain the absence of an effect for disgust in Study 1. This interpretation is consistent with the view that disgust is triggered by objects or behaviors appraised as impure [34] and research shows that feeling disgusted by moral violations of purity, such as unusual sexual practices, predicts harsher moral criticism of those practices [35]. However, the effect of experienced disgust on judgments of moral violations of purity is likely to be far more complex when considering the findings of Study 2.

Study 2 was designed to address some of the limitations of Study 1, including employing disgust and discomfort inductions with more comparable levels of intensity. Study 2 also examined the effects of the affect inductions on morality *and* punishment evaluations of purity and non-purity violations. Very little is known about differential effects of experienced disgust on judgments of moral severity compared to those of punishment. Furthermore, examination of potential differences between purity and non-purity violations is an important contribution to the literature given that the majority of the past research on disgust and moral judgment has either not tested or not found evidence of specificity (e.g., [1]). Contrary to predictions, no differences were observed for morality evaluations. However, participants who were randomized to experience disgust recommended harsher punishment for moral violations of purity (e.g., urinating on someone’s car door handle) relative to violations unrelated to purity (e.g., keying someone’s car). In contrast, those in the discomfort condition recommended more severe punishment for non-purity than purity violations. Although the effect was not especially robust, it is consistent with research by Rozin and colleagues [25] showing that violations of purity are most closely associated with disgust. This finding is also in line with previous research showing that disgust priming influenced moral evaluations regarding virtues and violations of purity, but not of fairness [6]. It is not immediately clear why experienced disgust would influence punishment judgments but not moral severity judgments. However, differences in judgements of punishment and morality have been observed in the literature. For example, Lieberman and Linke [36] found that a subject's relatedness to a transgressor affected reported punishment levels but not judgments of moral wrongness. In addition to being consistent with previous research, the present study extends previous research by directly comparing the experience of disgust to another form of negative affect while morality ratings were simultaneously obtained.

Evolutionary pressures may partially account for why disgust appears to have some input into moral judgments associated with purity violations [37]. There is now considerable evidence that disgust serves the evolved function of disease-avoidance [7]. Given that purity violations (engaging in consensual incest, receiving a blood transfusion from a child molester, eating rotten meat) often include direct or indirect contact with pathogens, it is perhaps not surprising that experiencing disgust is more likely to influence perceived purity violations compared to non-purity violations [38]. A recent review of the literature does suggest that moral transgressions genuinely evoke disgust, even when they do not reference physical disgust stimuli such as unusual sexual behaviors or the violation of purity norms [39]. Recent research also suggests that trait disgust is related to moral judgments outside of the purity domain [40]. Although violations unrelated to purity violations may evoke disgust, especially among those high in trait disgust proneness, experiencing disgust extraneously may have more of an influence on violation of purity norms relative to other norm violations.

The difference between punishment ratings of non-purity and purity violation in Study 2 was significantly different from zero for those in the disgust (*p* = .03) and discomfort (*p* = .04) condition but not for those in the neutral condition (*p* = .75). This suggests that punishment ratings for violations of purity and those of non-purity do not significantly differ for those in the ‘neutral’ affect condition. Although participants who experienced disgust recommended harsher punishment for moral violations of purity than for violations unrelated to purity, Table 3 shows that the magnitude of the punishment ratings for those in the neutral condition were comparable to those of participants in the disgust condition. This is consistent with those of Study 1, in which moral judgments in the disgust and neutral conditions did not significantly differ. The observed differences that are robust within the disgust induction group that are less robust when making comparisons with the neutral induction group is consistent with the recently articulated view that disgust may only marginally influence the severity of a very restricted category of moral violations [33]. This finding may also reflect unanticipated complexities of the neutral affect induction used in the present study. Although the induction validation data suggests that placing ones hand in lukewarm water does not evoke significant disgust or discomfort, it may very well not be entirely neutral. In fact, placing ones hand in lukewarm water may be cleansing. This observation highlights the importance of careful consideration of the nature of the comparison conditions employed in studies of this kind.

Although the findings of Study 2 suggest that an element of impurity may be required for disgust to influence judgments about such violations, it remains unclear if experiencing a cleanliness state that is opposite to disgust also influences moral judgments. Previous research suggests that a cleanliness induction immediately following a disgust induction lessens the severity of judgments about the misbehavior of others [5]. However, other research indicates that cleanliness inductions may also lead to increased severity of moral judgments [41]. Future research is needed to clarify these findings and to delineate if the demonstrated effect of reduced [5] or increased [41]. severity in moral judgments is specifically associated with feeling clean or feeling positive (which is associated with feeling clean).

Future research is also needed to clarify the mechanism that may account for the effects of experiencing disgust on morally-relevant judgments about purity violations. Research has shown that embodied gustatory experiences may affect moral processing [42]. Consistent with this view, a recent study found that reading about moral transgressions resulted in inducing gustatory disgust [43]. Purity violations may then have better access to gustatory processes that are readily accessed by disgust-relevant cues. Although the experience of disgust may be extraneous to the moral purity violation, the “matching” of the embodied experience between disgust and purity violations may lead one to be more prone to influencing the other. A shared embodied experience may then explain the association between disgust and morally-relevant judgments about purity violations. However, it is not clear that such a mechanism would necessarily lead to the conclusion that morality and experienced disgust share a common oral origin as previously proposed [2]. In fact, a recent study found that linguistic priming alone can transform a moral transgression into a viscerally repulsive event and that susceptibility to this priming varies as a function of an individual's sensitivity to the origins of visceral disgust [44].

Recent research has found that disgust proneness, as assessed with the DS-R, are significantly correlated with moral condemnation of social transgressions [40]. Although exploratory analysis found no link between the DS-R and ratings of moral severity in Study 1, scores on the DS-R were significantly correlate with morality ratings for purity offenses in Study 2. Of note is that Study 1 consisted of a relatively small number of moral violations that were in distinct categories of severity. In contrast, Study 2 consisted of more moral violations that were on a similar continuum of severity. Indeed, the immoral action in some of the violations (e.g., murder) was markedly different from others (e.g., assault). These exploratory findings suggest that nature of the moral violation, its severity, and how they are assessed may be important when considering the link between individual differences in disgust and moral judgments. Research along these lines highlights the need for more comprehensive models that can fully account for the complex association between state and trait disgust processes and moral decision-making.

Although the present findings contribute to the literature on the influence of experiencing disgust on morally-relevant judgments, limitations of the present investigation do warrant consideration before definitive inferences can be made. For example, it is unclear to what the experience of “discomfort,” which was experienced in the ice water condition, pertains. The ice water condition was employed as an alternative affective comparison condition given previous work suggesting that disgust can be evoked more readily and intensely than other negative basic emotions [45]. Given the nature of the ice water induction, it is possible that participant’s ratings of discomfort reflect physical pain. Indeed, cold pressor tasks have traditionally been used in the literature to assess pain [46]. Given research showing that pain and negative emotion share similar neurobiological substrates [47], delineating the characteristics of pain that influences moral decisions in future research may be informative. Prior research suggests that pain is often associated with aggression and a desire to punish [48]. This suggests that motivational states may be salient in how basic emotions influence moral judgments. Indeed recent research suggests that the effects of induced emotions on moral judgments can be predicted by taking their motivational dimension into account [49]. However, use of the experience of “discomfort” with ice water as a comparison to disgust may also be a viewed as a limitation of the present study. Indeed, the emotion of anger may be more directly relevant to moral decisions and should be the focus of research along these lines.

The results of Study 2 showed that those who had their hand submerged in ice water recommended harsher punishment for non-purity violations compared to purity violations. If motivational states do partially explain how emotions influence moral judgments, those of pain may have a stronger influence on non-purity violations compared to purity violations. Although discrepancies in the degree to which a target emotion is elicited is a common limitation in this line of research, future research where emotional features (i.e., arousal, valance, duration, intensity) are better controlled across a wider array of basic and complex emotions will contribute greatly to our understanding of the role of affect in moral decisions. Another limitation of the present investigation that should be considered with interpreting these findings is the relatively small samples sizes in Study 1 and Study 2. Replication with larger samples that afford more power in future research will offer greater confidence in the present findings.

## Appendix A

**Non-Morally Questionable Behaviors**
1. A.T. took a friend to the movies
2. D.H. went to lunch with an acquaintance
3. F.W. went for a walk in the park
4. L.E. bought a new lawnmower

**Moderate Morally Questionable Behaviors**
1. N.D. parked in a handicapped spot
2. T.R. neglected an elderly relative
3. K.D. lied to a good friend
4. Q.P. stole out of the collection plate at church

**Severe Morally Questionable Behaviors**
1. G.W. murdered 2 people in their own home
2. J.Q. raped a social worker
3. L.T. tortured someone with needles
4. R.Y. beat a homeless person unconscious

## Appendix B

**Non-Purity Violations**
1. John lied to a good friend
2. John spread unflattering rumors about a friend
3. John lied about how many hours he worked
4. John ran to knock someone off of a bike
5. John intentionally gave someone wrong directions
6. John cut up someone’s jacket
7. John scratched someone’s car with a key
8. John broke someone's window with a rock
9. John sold a customer a known-to-be defective product
10. John backed into someone’s car and didn’t leave a note

**Purity Violations**
1. John spit into someone’s drink
2. John knowingly served someone food past its expiration date
3. John forced someone to drink spoiled milk
4. John pushed someone into a dumpster which was swarming with cockroaches
5. John hazed members of his group by urinating on them
6. John urinated on someone’s car door handle
7. John threw someone’s cell phone into an unflushed toilet
8. John rubbed someone's toothbrush on the floor of a public restroom
9. John knowingly handled food at a restaurant immediately after using the bathroom
10. John intentionally aimed his vomit onto an air filter at a friend’s house

## References

[1] Wheatley T, Haidt J. Hypnotic disgust makes moral judgments more severe. Psychol Sci. 2005;16(10):780–4. 16181440 10.1111/j.1467-9280.2005.01614.x

[2] Chapman HA, Kim DA, Susskind JM, Anderson AK. In bad taste: Evidence from the oral origins of moral disgust. Science. 2009;323(5918):1222–6. 10.1126/science.1165565 19251631

[3] Rozin P, Haidt J, McCauley CR. (2000). Disgust In Lewis M, Haviland-Jones JM, editors. Handbook of emotions. New York: Guilford Press; 2000. p. 637–653.

[4] Haidt J. The moral emotions In: Davidson RJ, Scherer KR, Goldsmith HH, editors. Handbook of affective sciences. Oxford: Oxford University Press; 2003 p. 852–870.

[5] Schnall S, Benton J, Harvey S. With a clean conscience: Cleanliness reduces the severity of moral judgments. Psychol Sci. 2008;19(12):1219–22. 10.1111/j.1467-9280.2008.02227.x 19121126

[6] Horberg EJ, Oveis C, Keltner D, Cohen AB. Disgust and the moralization of purity. J Pers Soc Psychol. 2009;97(6):963–76. 10.1037/a0017423 19968413

[7] Oaten M, Stevenson RJ, Case TI. Disgust as a disease-avoidance mechanism. Psychol Bull. 2009;135(2):303–21. 10.1037/a0014823 19254082

[8] Miller WI. The anatomy of disgust. Cambridge, MA: Harvard University Press; 1997.

[9] Peters E, Vastfjall D, Garling T, Slovic P. Affect and decision making: A "hot" topic. J Behav Decis Making. 2006;19(2):79–85.

[10] Schnall S, Haidt J, Clore GL, Jordan AH. Disgust as embodied moral judgment. Pers Soc Psychol Bull. 2008;34(8):1096–1109. 10.1177/0146167208317771 18505801 PMC2562923

[11] Moretti L, di Pellegrino G. Disgust selectively modulates reciprocal fairness in economic interactions. Emotion. 2010;10(2):169–80. 10.1037/a0017826 20364893

[12] Haidt J, Graham J. When morality opposes justice: Conservatives have moral intuitions that liberals may not recognize. Soc Justice Res. 2007;20(1):98–116.

[13] Haidt J, Koller S, Dias M. Affect, culture, and morality, or is it wrong to eat your dog? J Pers Soc Psychol. 1993;65(4):613–628. 8229648 10.1037//0022-3514.65.4.613

[14] Royzman E, Atanasov P, Landy JF, Parks A, Gepty A. CAD or MAD? Anger (not disgust) as the predominant response to pathogen-free violations of the divinity code. Emotion. 2014;14(5):892–907. 10.1037/a0036829 24866519

[15] Olatunji BO, Forsyth JP, Cherian A. Evaluative differential conditioning of disgust: A sticky form of relational learning that is resistant to extinction. J Anxiety Disord. 2007;21(6):820–34. 17158024 10.1016/j.janxdis.2006.11.004

[16] Scherer KR, Wallbott HG. Evidence for universality and cultural variation of differential emotion response patterning. J Pers Soc Psychol. 1994;66(2);310–328. 8195988 10.1037//0022-3514.66.2.310

[17] Fiedler K, Nickel S, Muehlfriedel T, Unkelbach C. Is mood congruency and effect of genuine memory or response bias? J Exp Soc Psychol. 2001;37(3):201–214.

[18] Gilboa E, Roberts JE, Gotlib IH. The effects of induced and naturally occurring dysphoric mood on biases in self-evaluation and memory. Cognition Emotion. 1997;11(1):65–82.

[19] Chepenik LG, Cornew LA, Farah MJ. The influence of sad mood on cognition. Emotion. 2007;7(4):802–811. 18039049 10.1037/1528-3542.7.4.802

[20] Bodenhausen GV, Gabriel S, Lineberger M. Sadness and susceptibility to judgmental bias: the case of anchoring. Psychol Sci. 2000;11(4):320–323. 11273392 10.1111/1467-9280.00263

[21] Haidt J, McCauley C, Rozin P. Individual differences in sensitivity to disgust: A scale sampling seven domains of disgust elicitors. Pers Indiv Differ. 1994;16(5):701–713.

[22] Olatunji BO, Williams NL, Tolin DF, Abramowitz JS, Sawchuk CN, Lohr JM, et al The disgust scale: Item analysis, factor structure, and suggestions for refinement. Psychol Assess. 2007;19(3):281–297. 17845120 10.1037/1040-3590.19.3.281

[23] Tsao SD, McKay D. Behavioral avoidance tests and disgust in contamination fears: distinctions from trait anxiety. Behav Res Ther. 2004;42(2):207–16. 14975781 10.1016/S0005-7967(03)00119-0

[24] Lindquist K, Barrett LF. Emotional complexity In Lewis M, Haviland-Jones LM, Barrett LF. The handbook of emotion. New York: Guilford; 2008 p. 513–530.

[25] Rozin P, Lowery L, Imada S, Haidt J. The CAD triad hypothesis: A mapping between three moral emotions (contempt, anger, disgust) and three moral codes (community, autonomy, divinity). J Pers Soc Psychol. 1999;76,(4):574–586. 10234846 10.1037//0022-3514.76.4.574

[26] Hutcherson CA, Gross JJ. The moral emotions: A social-functionalist account of anger, disgust, and contempt. J Pers Soc Psychol. 2011;100(4):719–737. 10.1037/a0022408 21280963

[27] Olatunji BO, Puncochar BD. Delineating the influence of emotion and reason on morality and punishment. Rev Gen Psychol. In press.

[28] Haidt J. The emotional dog and its rational tail: A social intuitionist approach to moral judgment. Psychol Rev. 2001;108(4):814–834. 11699120 10.1037/0033-295x.108.4.814

[29] Buckholtz JW, Asplund CL, Dux PE, Zald DH, Gore JC, Jones OD, et al The neural correlates of third-party punishment. Neuron. 2008;60(5):930–940. 10.1016/j.neuron.2008.10.016 19081385

[30] Kurzban R, DeScioli P, O'Brien E. Audience effects on moralistic punishment. Evol Hum Behav. 2007;28(2):75–84.

[31] Alicke MD. Culpable causation. J Pers Soc Psychol. 1992;63(3):368–378.

[32] Schwarz N, Clore GL. Mood, misattribution, and judgments of well-being: Informative and directive functions of affective states. J Pers Soc Psychol. 1983;45(3):513–523.

[33] May J. Does disgust influence moral judgment? Australas J Philos. 2014;92(1);124–141.

[34] Horberg EJ, Oveis C, Keltner D. Emotions as moral amplifiers: An appraisal tendency approach to the influences of distinct emotions upon moral judgment. Emotion Rev. 2011;3(3):237–244.

[35] Haidt J, Hersh MA. Sexual morality: The cultures and emotions of conservatives and liberals. J App Soc Psychol. 2001;31(1):191–221.

[36] Lieberman D, Linke L. The effect of social category on third party punishment. Evol Psychol. 2007;5(2);291–307.

[37] Tybur JM, Lieberman D, Kurzban R, DeScioli P. Disgust: Evolved function and structure. Psychol Rev. 2013;120(1):65–84. 10.1037/a0030778 23205888

[38] Inbar Y, Pizarro DA, Bloom P. Disgusting smells cause decreased liking of gay men. Emotion. 2012;12(1):23–27. 10.1037/a0023984 21707161

[39] Chapman HA, Anderson AK. Things rank and gross in nature: A review and synthesis of moral disgust. Psychological Bull. 2013;139(2):300–327. 10.1037/a0030964 23458435

[40] Chapman HA, Anderson AK. Trait disgust is related to moral judgments outside of the purity domain. Emotion. 2014;14(2)341–348. 24512243 10.1037/a0035120

[41] Zhong CB, Strejcek B, Sivanathan N. A clean self can render harsh moral judgment. J Exp Soc Psychol. 2010;46(5):859–862.

[42] Eskine KJ, Kacinik NA, Prinz JJ. A bad taste in the mouth: Gustatory disgust influences moral judgment. Psychol Science. 2011;22(3):295–299. 10.1177/0956797611398497 21307274

[43] Eskine KJ, Kacinik NA, Webster GD. The bitter truth about morality: Virtue, not vice, makes a bland beverage taste nice. PLoS ONE. 2012;7(7):e41159 10.1371/journal.pone.0041159 22815953 PMC3399822

[44] Herz RS. Verbal priming and taste sensitivity make moral transgressions gross. Behav Neurosci. 2014;128(1):20–28. 10.1037/a0035468 24512062

[45] Gross JJ, Levenson RW. Emotion elicitation using films. Cognition Emotion. 1995;9(1):87–108.

[46] Helsen K, Goubert L, Peters ML, Vlaeyen JWS. Observational learning and pain-related fear: An experimental study with colored cold pressor tasks. J Pain. 2011;12(12):1230–1239. 10.1016/j.jpain.2011.07.002 22019133

[47] Buhle J, Kober H, Ochsner KN, Mende-Siedlecki P, Weber J, Hughes B, et al Common representation of pain and negative emotion in the midbrain periaqueductal gray. Soc Cogn Affect Neurosci. 2012;8(6):609–616. 10.1093/scan/nss038 22446299 PMC3739905

[48] Berkowitz L. Pain and aggression: Some findings and implications. Motiv Emotion. 1993;17(3):277–293.

[49] Ugazio G, Lamm C, Singer T. The role of emotions for moral judgments depends on the type of emotion and moral scenario. Emotion. 2012;12(3):579–590. 10.1037/a0024611 21859189

